# Beyond the Nucleus: Plastic Chemicals Activate G Protein-Coupled Receptors

**DOI:** 10.1021/acs.est.3c08392

**Published:** 2024-03-05

**Authors:** Molly McPartland, Sarah Stevens, Zdenka Bartosova, Ingrid Gisnås Vardeberg, Johannes Völker, Martin Wagner

**Affiliations:** Department of Biology, Norwegian University of Science and Technology (NTNU), Trondheim 7491, Norway

**Keywords:** endocrine disrupting chemicals, plastics, GPCRs, cell surface receptor, signaling disruption, screen, PRESTO-Tango, food packaging

## Abstract

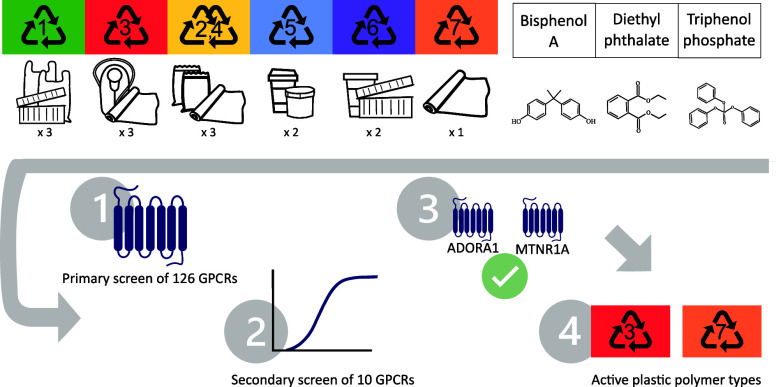

G protein-coupled receptors (GPCRs) are central mediators of cell signaling and physiological function. Despite their biological significance, GPCRs have not been widely studied in the field of toxicology. Herein, we investigated these receptors as novel targets of plastic chemicals using a high-throughput drug screening assay with 126 human non-olfactory GPCRs. In a first-pass screen, we tested the activity of triphenol phosphate, bisphenol A, and diethyl phthalate, as well as three real-world mixtures of chemicals extracted from plastic food packaging covering all major polymer types. We found 11 GPCR-chemical interactions, of which the chemical mixtures exhibited the most robust activity at adenosine receptor 1 (ADORA1) and melatonin receptor 1 (MTNR1A). We further confirm that polyvinyl chloride and polyurethane products contain ADORA1 or MTNRA1 agonists using a confirmatory secondary screen and pharmacological knockdown experiments. Finally, an analysis of the associated gene ontology terms suggests that ADORA1 and MTNR1A activation may be linked to downstream effects on circadian and metabolic processes. This work highlights that signaling disruption caused by plastic chemicals is broader than that previously believed and demonstrates the relevance of nongenomic pathways, which have, thus far, remained unexplored.

## Abstract 2

Plastics are chemically complex materials. This work demonstrates that plastic chemicals can interfere with cell surface receptors, providing a deeper understanding of the mechanisms by which chemical exposure can contribute to noncommunicable diseases.

## 1 Introduction

G protein-coupled receptors (GPCRs) are the largest class of cell surface receptors and transduce signals from a diverse array of ligands across the cell membrane. They function as central regulators for most cellular processes, including cell differentiation, migration, apoptosis, and growth.^1^ Physiologically, disruption of GPCR signaling is linked to many diseases, underscored by the fact that nearly half of all pharmaceuticals target GPCRs.^2^ Despite their biological significance, GPCRs have received very little attention in toxicological research. This is striking given that endocrine-disrupting chemicals (EDCs), compounds that can “interfere with any aspect of hormone action,”^3^ often act via cellular receptors and represent a major topic of research.

Indeed, emerging evidence suggests that EDCs or synthetic chemicals can interact with several rhodopsin-like GPCRs as well. A prominent example is bisphenol A (BPA) activating G protein-coupled estrogen receptor 1 (GPER), leading to various cancer-promoting mechanisms, such as increased reactive oxygen species,^4^ cellular proliferation,^5^ apoptosis,^6^ and cell migration.^7^ Beyond GPER and bisphenols, scattered evidence suggests that other chemicals can also interfere with GPCR signaling. For instance, *p*,*p*′-DDT allosterically activates the human follitropin receptor (FSHR),^8^ multiple phthalate esters inhibit the cannabinoid-1 (CB_1_) receptor,^9^ and certain carbamate insecticides activate or inhibit melatonin receptors.^10,11^ Furthermore, GPCRs, specifically the angiotensin (AT1), dopamine (D2), adrenergic (β1), acetylcholine (M1), and histamine (H1) receptors, have been used in the monitoring of pharmaceuticals in wastewater.^12,13^ While not comprehensive, these reports demonstrate that GPCRs are susceptible to known EDCs and other synthetic chemicals.

Plastics are a common source of human exposure to chemicals, among them EDCs.^14−16^ Well-studied plastic chemicals, such as BPA, have been detected in over 90% of US, European, and Asian populations.^17^ Such exposure is linked to an increased prevalence of noncommunicable diseases including asthma, obesity and diabetes, hormone-sensitive carcinogenesis, and impaired immune function.^18^ The associated health costs of exposure to plastic-associated EDCs are estimated to be 56 billion US dollars annually. This shows that certain plastic chemicals significantly contribute to the burden of disease.

Plastics can contain thousands of chemicals in addition to phthalates and BPA.^19^ This includes both intentionally added substances, such as plasticizers, colorants, stabilizers, and flame retardants,^20^ and nonintentionally added substances (NIAS), such as unreacted monomers, reaction byproducts, degradation products, and impurities.^21^ As these chemicals are not covalently bound to the polymer, they can leach into liquids, solids, or air via migration or volatilization, resulting in human exposure to both known and unknown chemicals. Thus, realistic exposure scenarios must consider complex mixtures of plastic chemicals. Assessing toxicity of the overall mixture of compounds released by plastics encompasses all exposure-relevant chemicals and is key to understanding their joint impacts and hazards.^22^

In contrast to the dedicated research on plastic chemicals mediating their toxicity via nuclear receptors, an interrogation of such chemicals across human GPCRs has not yet been undertaken. Given that there are ∼400 non-olfactory GPCRs that may, in principle, be targeted by plastic chemicals, high-throughput screening of many GPCRs represents an ideal approach. To this end, we adapted the PRESTO-Tango (parallel receptorome expression and screening via transcriptional output, with transcriptional activation following beta-arrestin translocation) platform used in drug development^23^ to investigate whether plastic chemicals activate non-olfactory GPCRs. To address the chemical complexity of plastic products, including unknown substances and potential mixture toxicity, we evaluated GPCR agonism caused by all extractable chemicals in plastic food contact articles (FCAs), in addition to individual, well-known chemicals used in plastics.

In this work, we aimed to investigate (i) whether chemicals present in plastic FCA activate specific GPCRs, (ii) whether certain polymer types or products contain such GPCR agonists, and (iii) the potential biological implications of GPCR activation. Here, we identify several novel receptor–chemical interactions that were confirmed via dose-dependent activation and pharmacological knock-down using known GPCR antagonists. We further identify specific polymer types and products containing GPCR agonists. Finally, we identify biological processes associated with the respective GPCR disruption.

## 2 Materials and Methods

### 2.1 Rationale and Study Design

In contrast to the conventional method of testing multiple samples on a single target, we utilized the PRESTO-Tango platform to assess many GPCRs across a smaller set of samples. Accordingly, we screened three individual plastic chemicals and three chemical mixes derived by combining several plastic extracts. We applied a tiered approach starting with a first-pass primary screen to identify GPCRs activated by the samples. We then employed a secondary screen to confirm the dose dependence of these GPCR–chemical interactions. In the third step, we tested the individual plastic FCA extracts previously used to create the mixes to determine which specific products and polymer types contain GPCR agonists. Lastly, pharmacological knockdown of the GPCR activity served as the final step of confirmation.

For the primary screen, we selected BPA (CAS: 80-05-7, 99.5%, Sigma-Aldrich), diethyl phthalate (DEP, 84-66-2, 99.5%, Sigma-Aldrich), and triphenol phosphate (TPP, 115-86-6, >99%, Sigma-Aldrich) because they are (1) present in plastic FCAs, (2) classified as EDCs,^15,19^ and (3) detected in more than 50% of the examined human populations.^24^ To address “real-world” chemicals present in plastic FCAs, we extracted everyday plastic food packaging items using methanol (see Section 2.2). Specifically, we selected the plastic types with the highest global production volumes^25^ (polypropylene (PP), polyethylene (PE), polyethylene terephthalate (PET), polystyrene (PS), polyvinyl chloride (PVC), polyurethane (PUR)) from the four countries with the highest volume of plastic waste per capita (USA, Germany, England, South Korea),^26^ as well as from local grocery stores and suppliers in Norway. We pooled three products made of PET to make the PET mix and three products made of polyvinyl chloride (PVC mix). We focused on these polymer types because of their weak and strong respective toxicity at a range of endpoints.^27^ The third mix (assorted mix) contained three PE products, two PP products, two PS products, and one PUR product (Table 1 and Figure S1). Each mix contained FCAs originating from at least two differing countries.

**Table 1:** Composition of the Mixes of Plastic Extracts Screened in this Study and Results from Nontarget Chemical Analysis (Number of Chemical Features Detected)

sample name	sample component	country of origin	product	dilution in mix	no. of features	unique features
PET mix	PET 1	Germany	oven bag	1:3	521	846
	PET 2	UK	oven bag	1:3	96	
	PET 3	Norway	food container	1:3	292	
PVC mix	PVC 1	Germany	drinking tube	1:3	1374	4222
	PVC 2	Germany	drinking tube	1:3	700	
	PVC 3	UK	cling film	1:3	3804	
assorted mix	PE 1	Germany	zip lock freezer bag	1:8	1202	5578
	PE 2	S. Korea	freezer bag	1:8	229	
	PE 3	USA	cling film	1:8	356	
	PP 1	S. Korea	coffee cup	1:8	642	
	PP 2	USA	yoghurt cup lid	1:8	990	
	PS 1	USA	cup^a^	1:8	1534	
	PS 2	Norway	tray^a^	1:8	526	
	PUR 1	Germany	hydration bladder	1:64	3050	

a Extruded polystyrene, S. Korea = South Korea, UK = United Kingdom of Great Britain and Northern Ireland, USA = United States of America.

With this mixture design and stepwise confirmation, we optimized the number of plastic products we could test and enabled comparison between and within single plastic chemicals (BPA, DEP, and TPP) and the real-world chemical mixtures present in plastic products.

### 2.2 Sample Selection and Preparation

A single solvent-based extraction was performed for all samples, with each of the 14 extracts being individually produced as described in depth elsewhere^28^ and subsequently combined to produce the mixes. Methanol was used as the solvent because it does not dissolve or swell the polymer types included here and thereby represents a more realistic, albeit accelerated, migration scenario. Briefly, 13.5 g of plastic of each sample was extracted in 90 mL of methanol (99.8%, Sigma-Aldrich) by sonification for 1 h. Without evaporating to dryness, we removed a 60 mL aliquot from the 90 mL extract, added 600 μL of dimethyl sulfoxide (DMSO), and evaporated the samples under a gentle stream of nitrogen to a final volume of 600 μL. Three procedural blanks were included to control for potential contamination (Figure S2). The PVC and PET mixes were then made by combining equal volumes of the three PVC and PET extracts. The assorted mix was made similarly with the combination of eight extracts; however, PUR 1 was diluted 1:8 in DMSO due to its cytotoxicity. We present the concentration of plastic as mg plastic well^–1^, which corresponds to the chemicals extracted from that mass of plastic dissolved and analyzed in 60 μL of cell culture media per well.

### 2.3 Nontarget Chemical Analysis

We performed a nontarget chemical analysis of the plastic extracts as part of a previous study with the complete methods detailed there (corresponding sample names in Table S1).^28^ In brief, we analyzed each sample on a Waters Acquity UPLC I-Class coupled to a quadrupole time-of-flight mass spectrometer (Synapt G2-S HDMS, Waters) run in positive electron spray ionization mode and performed the data analysis using Progenesis QI (Metascope algorithm, Nonlinear Diagnostics) as previously reported.^28^

Since we used a smaller sample set than before,^28^ a realignment of the mass spectra allowed us to process all samples together and created a joint list of chemical features (i.e., ions with a unique *m*/*z* and retention time). Only features that were absent in the procedural blanks or had at least 10-fold higher raw abundances were included.

### 2.4 Cell Care and Plasmid Isolation

HTLA cells, HEK293 cells stably expressing a tTA-dependent luciferase reporter, and a β-arrestin-TEV protease fusion gene (kindly provided by Brian Roth) were maintained in DMEM (Sigma-Aldrich, D6492), supplemented with 10% FBS (Sigma-Aldrich, F9665), 2 μg mL^–1^ puromycin (58-58-2, ≥98%, Invitrogen), and 100 μg mL^–1^ hygromycin (31282-04-9, ≥98%, Invitrogen). Cells were split 1:5 when confluency reached 70% and discarded after passage 10. All GPCR plasmids originated from a Roth lab PRESTO-Tango kit (Addgene, Kit #1000000068) as *E. coli* glycerol stocks. Each stock was individually cultured, and plasmids were isolated via minipreps (Promega, A1330). Each GPCR plasmid concentration was quantified (Nanodrop, One/One^c^ microvolume UV–vis spectrophotometer, ND-ONE-W, Thermo Scientific) and had a 260/280 ratio of 1.7–1.9, a 260/230 ratio of ≥1.8, and concentrations of ≥100 ng/μL. Plasmid stocks for each GPCR were then diluted for a final concentration of 50 ng μL^–1^.

### 2.5 Selection of GPCRs for Primary Screen

While the PRESTO-Tango assay is designed for simultaneous screening of 315 non-olfactory GPCRs, we excluded 167 of those receptors that could not be validated with an agonist in the original publication of the assay.^23^ Without such validation, we cannot differentiate false negatives from true negatives because these receptors lack functioning reference compounds that could serve as positive control. An additional 22 of the validated receptors were excluded, because amplification did not produce sufficient plasmid quantity or quality. A total of 126 GPCRs were thus included in this study (Table S2).

### 2.6 Transfection Efficiency and Immunostaining

Prior to the screen, we optimized transfection efficiency based on number of seeded cells, volumes of transfection reagents, and plasmid concentrations using six GPCRs (arginine vasopressin receptor 1A (AVPR1A), arginine vasopressin receptor 1A (AVPR1B), arginine vasopressin receptor 2 (AVPR2), human copper transporter 1 (HCTR1), melatonin receptor 1A (MTNR1A), melatonin receptor 1B (MTNR1B)). We selected these receptors due to their range of constitutive activity and differences in natural ligands (small molecule vs peptide hormone) (Figure S3). Transfected HTLA cells were fixed (4% paraformaldehyde, Thermo Scientific, 28908), quenched (0.1 M ammonium chloride, ≥99.5%, Sigma-Aldrich, A9434), permeabilized (0.5% Triton-x, Sigma-Aldrich, X100), and blocked, all at room temperature. Cells were incubated first with anti-Flag antibody (1:500, polyclonal mouse anti-Flag, Sigma-Aldrich, F1804) and then with Alexa Fluor 594-conjugated goat antimouse antibody (1:200, Invitrogen, A32742) and nuclear dye (Fisher Scientific, R37605). Cells were washed with PBS (0.5 mM CaCl_2_, pH 7.4), sealed, and stored until imaging. Two images per field (NucBlue and Alexa Fluor) and two fields per well were captured on a Cytation 5 cell imaging multimode reader (BioTek, details in Supporting Methods and Materials). Images were analyzed using the open source software CellProfiler^29^ (Supporting Methods and Materials). Transfection efficacy was calculated as a ratio between the number of transfected cells and the total number of cells in the well.

### 2.7 Cell Viability

Cell viability was measured using the nuclei count data (NucBlue staining) in nontransfected HTLA cells. We defined cytotoxic effects as a 20% reduction of cell viability compared to the controls. Briefly, cells were seeded at a density of 10 000 cells well^–1^ in white, optical bottom 384-well plates (Sigma-Aldrich, CLS3765) coated with 0.1 mg mL^–1^ poly-l-lysine (Sigma-Aldrich, P2636). The following day, cells were starved for 1 h in starving media (DMEM supplemented with 1% dialyzed fetal bovine serum (dFBS, Thermo Fisher, A3382001), 1× penicillin/streptomycin) before addition of BPA, DEP, TPP, plastic extracts, and the three mixes. The exposure lasted 23 h before being stained with NucBlue and imaged as described above. To confirm that cell viability was similar between transfected cells, we compared nontransfected cells to those expressing MTNR1B and AVPR2. No differences were observed at concentrations relevant to the screen (Figure S4), and results were generalized to all receptors.

### 2.8 PRESTO-Tango Assay

Both the primary and secondary screens were performed as previously described^30^ with several notable modifications. On day 1, cells were seeded as described in Section 2.7 and transfected (50 ng plasmid well^–1^) 24 h later (day 2) using Lipofectamine 3000 following the supplier’s instructions (ThermoFisher, L3000008). On day 3, the transfection media were removed and replaced with starving media, and after 1 h of starvation, the chemicals and mixes were diluted in starving media and added to the cells (described in the Supporting Information). The exposure lasted for 23 h before cells were lysed, and quantification of luminescence was done on a Cytation 5 cell imaging multimode reader (BioTek).

For the primary screen, the exposure and transfection layout were such that each plate contained ten receptors exposed to 10 μM BPA, DEP, or TPP as well as 0.9 mg plastic well^–1^ of the PVC, PET, or assorted mix, a negative control (starving media only), and solvent control (0.2% DMSO). Additionally, cells transfected with MTNR1B and exposed to melatonin (≥98%, Sigma-Aldrich, M5250) served as a positive control on each plate while nonexposed, nontransfected cells served as background (Figure S5). Each control and treatment were analyzed in four technical replicates (i.e., wells) in a single experiment. For the secondary screen, we constructed seven-point dose–response curves from a 1:2 dilution series with the highest concentration being 30 μM for the single chemicals and 1.8 mg plastic well^–1^ for the mixes. All secondary screen experiments included four technical replicates and three biological replicates (i.e., independent experiments).

### 2.9 Activity of Individual Plastic Extracts

We further investigated which of the FCAs constituting the active mixes caused the GPCR agonism observed in the primary and secondary screens. Therefore, we analyzed the three extracts used in the PVC mix and the eight extracts used in the assorted mix in the PRESTO-Tango assay for adenosine receptor 1 (ADORA1) and MTNR1A as described above (Section 2.8), with some modifications. On each plate, we now used 5′-*N*-ethylcarboxamidoadenosine (NECA, Abcam, ab120440) and melatonin as positive controls for ADORA1 and MTNR1A, respectively (Figure S6). A negative control (*n* = 8) and a solvent control (highest solvent concentration = 0.2%) constructed with a five point 1:2 dilution series (*n* = 4 each) were also included on each plate. The dilution of DMSO corresponds to that of the samples to ensure that there is no effect of DMSO.

### 2.10 Confirmation Using Pharmacological Knock-Down

To further confirm that the observed activation was indeed caused by agonism at the respective GPCR, we coexposed cells to the active samples and to the ADORA1 antagonist 8-cyclopentyl-1,3-dipropylxanthine (DCPCX, 99.02%, MedChemExpress, HY-100937)^31^ or the MTNR1A antagonist luzindole (97%, ThermoFisher, J61915-#0).^32^ We used the EC_80_ of NECA (0.05 μM) and of melatonin (0.01 μM) as the background agonist for DCPCX and luzindole, respectively. Based on the dose–response relationship of the antagonists, we selected three concentrations of DCPCX (8, 32, and 2000 pM) and luzindole (0.1, 1, and 10 μM). Cells transfected with ADORA1 or MTNR1A were then exposed to 1.8 mg plastic well^–1^ of PUR 1, PVC 1, PVC 2, and PVC 3 in combination with three concentrations of the respective antagonist (Supporting Methods and Materials).

### 2.11 Gene Ontology Analysis

Using AmiGO2,^33,34^ we extracted all gene ontology (GO) terms annotated to ADORA1 or MTNR1A. We filtered for terms specific to biological processes in mammals. We prioritized and ordered the GO terms using a structure-based ranking with the R package “GOxploreR.”^35^ Briefly, we removed redundant GO terms that were within the same GO directed acyclic graphs (GO–DAGs) (function “prioritizedGOTerms”) and ordered the remaining terms (function “scoreRankingGO”). The provided score (*s*_t_) was then used to rank terms based on biological specificity. In this manner, we emphasize biological processes in which the effects of ADORA1 and MTNR1A disruption may be better understood.

### 2.12 Data Analysis and Quality Control

All data and statistical analyses were conducted in R (R Core Team, 2022) or GraphPad Prism (v10, GraphPad Software, San Diego, CA). Data visualization and clustering were performed in R with “pheatmap”, “eulerr”, and “dendsort” packages. The dose–response curves were fit with the “drc” package using a four parameter logistic function (lower limit, upper limit, slope, EC_20_).^36^ For the cytotoxicity of the individual extracts and chemicals, the upper limit was constrained to 100, and the lower limit was constrained to 0. For all other curves, there was no upper or lower limit constraint.

The solvent and negative controls for the primary screen with all GPCRs were not significantly different (Wilcox–Mann–Whitney test, *p* > 0.05) and were pooled as a measure of constitutive activity (CA). The fold activation was calculated as luminescence of exposed cells (*n* = 4) divided by CA (*n* = 8). For the experiments with the individual plastic extracts and the antagonist assay, luminescence was normalized to CA (0%) and the upper limit of the positive control (100%). *z*-Scores were calculated to account for the standard deviation within replicates and accurately represent depression of activity (eq S1). A centroid hierarchical cluster analysis of all GPCRs, chemicals, and mixes was preformed using the *z*-scores from the primary screen in the “pheatmap” package.

As there is no defined threshold in the literature of what is considered a hit,^23,30^ we calculated the limit of detection (LOD) for each receptor as three times the standard deviation (SD) plus the mean of the CA (*n* = 8). We considered a receptor activation >LOD as a hit that warranted further investigation. For the secondary screen, we calculated the LOD from three biological replicates. For the recovery assay, one-way ANOVA was performed in GraphPad Prism.

Quality criteria were applied to the PRESTO-Tango results based on the data obtained from the negative, solvent, and positive controls on each plate. Stricter quality criteria for the secondary screen and antagonist experiments were employed for quality assurance of data (Supporting Methods and Materials and Table S3).

## 3 Results and Discussion

In this work, we performed the first large-scale screen to determine whether plastic chemicals can disrupt GPCR signaling by acting as agonists. We found that FCAs made of PVC and PUR contained potent activators of ADORA1 and MTNR1A receptors and confirmed the specificity of these novel receptor–chemical interactions using known GPCR antagonists. Finally, we identified biological processes involved in the targeted GPCRs and discussed their potential implications for human health.

### 3.1 Plastic Food Packaging Contains Thousands of Chemicals

Prior to the primary screen, we investigated the number of chemical features present in the plastic extracts using nontarget high-resolution mass spectrometry. Additionally, cytotoxicity of the samples was investigated to determine noncytotoxic concentrations to be used in the primary screen. An in-depth analysis of the chemical composition and tentative identification of chemicals in the present samples can be found in Stevens et al.^28^

In total, we detected 10 646 chemical features across all extracts (Table 1). In the individual samples, the number of features ranged from 96 (PET 2, oven bags) to 3804 (PVC 3, cling film). Of the three plastic mixes, the assorted mix contained the most features (5578), with 54% originating from PUR 1 (hydration bladder, 3050). PUR 1 was also the most cytotoxic extract (EC_20_ = 0.05 mg plastic well^–1^) and, thus, had to be diluted by an additional factor of 1:8 in the assorted mix. The other seven extracts in that mix contained fewer features, and their cytotoxicity was not associated with a specific polymer type or the number of features (Figure S7A). The PVC mix contained 4222 chemical features, with 90% coming from PVC 3 (cling film), which was also the most cytotoxic PVC extract (EC_20_ = 2.8 mg plastic well^–1^). The PET mix contained 846 features, with large differences between similar products (PET 1 = 521 vs PET 2 = 96). Despite these differences, the cytotoxicities of all three PET products were similarly low. As expected, the mixes were more cytotoxic than their individual components. As each individual component was diluted, this is likely due to the larger numbers of chemical features resulting from combining the extracts. Indeed, cytotoxicity increases with the number of chemicals in a sample.^37^ Congruent with previous reports,^27,38,39^ our results demonstrate the presence of a large number of chemicals in plastic FCAs that induce cytotoxicity, particularly in case of PVC and PUR products.

Of the single plastic chemicals included in this study, DEP (EC_20_ = 12.8 μM) and TPP (EC_20_ = 15.0 μM) were most cytotoxic (Figure S7B). BPA (EC_20_ = 34.6 μM) was not cytotoxic up to 30 μM; however at higher concentrations it also showed similar suppression of cell viability to that of DEP and TPP (Figure S8D).

### 3.2 Plastic Chemicals Activate GPCRs

**Figure 1:**
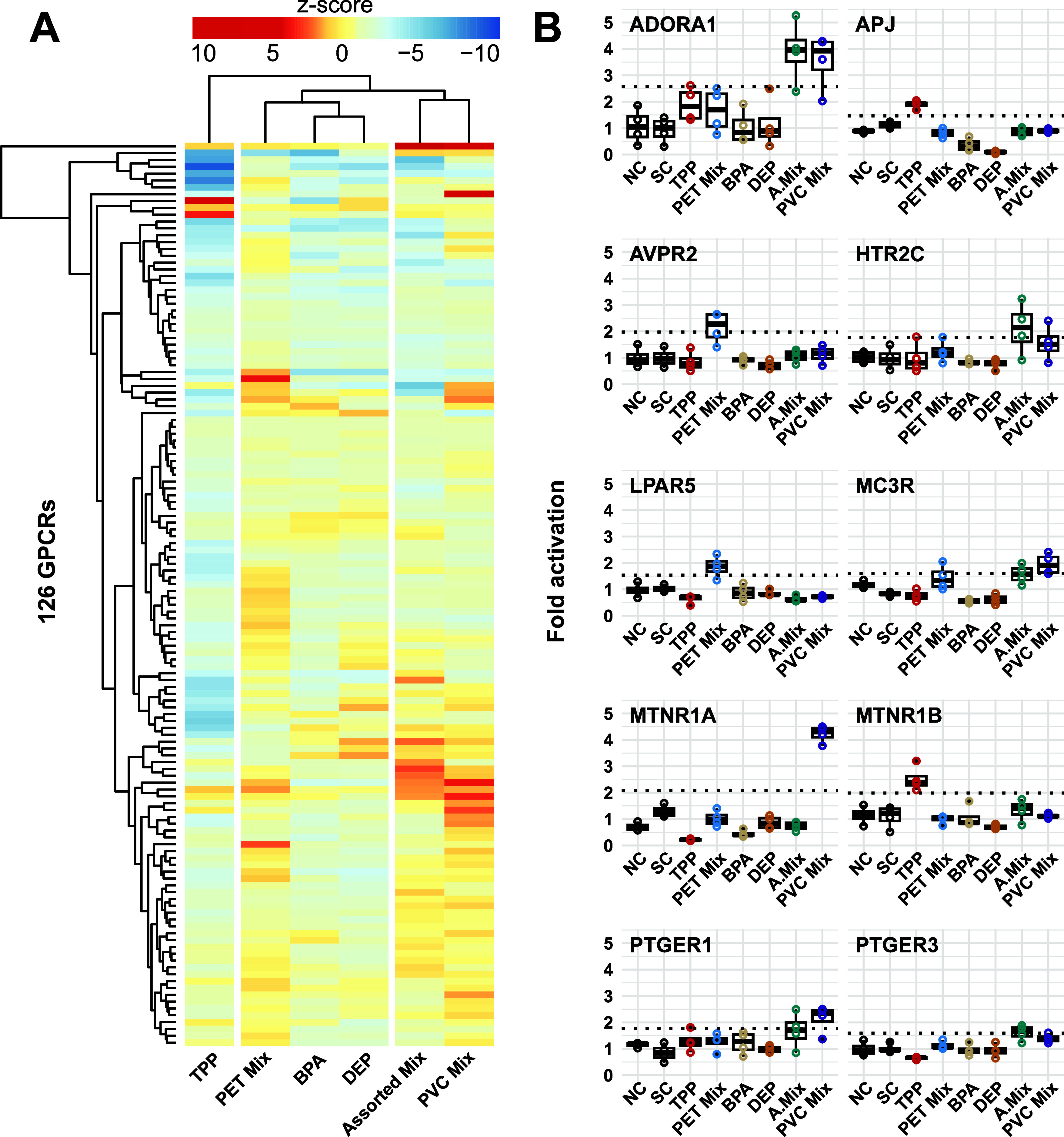
Results of the primary PRESTO-Tango screen of 126 GPCRs exposed to triphenol phosphate (TPP), PET mix, bisphenol A (BPA), diethyl phthalate (DEP), assorted mix, and PVC mix. (A) Heatmap displaying *z*-scores where red and blue indicate receptor activation and suppression, respectively. GPCRs, chemicals, and mixes are clustered based on activity. (B) Individual hits from primary screen were determined based on a receptor activation >LOD (dotted black line), the inactive chemicals and mixes are presented for comparison. NC = negative control, SC = solvent controls, *n* = 4 (technical replicates).

The primary screen was used as a “first pass” to identify receptor–chemical interactions. In total, we tested 756 potential interactions and found 11 hits on GPCRs from the adenosine, melatonin, apelin, lysophospholipid, melanocortin, prostanoid, 5-hydroxytryptamine, and vasopressin families (Figure 1A). These hits activated the respective receptor with a fold change of 1.61–4.22 and *z*-scores of 3.04–8.88 (Table S4). Unsurprisingly, hits were more prevalent and active across the mixes than in the single chemicals (Figure 1A). The PVC mix activated four receptors and was most effective on MTNR1A, followed by the assorted mix on ADORA1. None of the procedural blanks induced cytotoxicity or activated any of the GPCRs indicating that the sample preparation did not result in contamination with GPCR agonists (Figure S2).

In contrast, TPP inhibited the activity of 89% of the GPCRs (Figure 1A). Although not universal, such broad reduction in luminescence points toward a generic inhibitory effect rather than inverse agonism. Both compound aggregation and inhibition of the luciferase reporter are well-known issues in drug screening that increase the probability of false-negative hits.^40^ Indeed, previous work with the PRESTO-Tango assay has reported numerous compounds with similar promiscuous inhibitory activity.^23^ Screening for luciferase inhibition and inverse agonism is required to clarify the inhibitory effects of TPP. Despite this inhibition, TPP activated MTNR1B (fold activation = 2.52, *z*-score = 4.32), suggesting that TPP may be a more effective MTNR1B agonist than we report.

BPA and DEP did not produce a hit at any of the 126 GPCRs included in the primary screen. This is somewhat contrary to our expectations as radioligand binding assays have demonstrated that BPA activates ADORA1, dopamine receptor 1 (DRD1), and serotonin receptor 2C (5HT2C).^41^ GPER activation by BPA has also been well established,^5−7,42^ but given that GPER was not validated with an agonist in the PRESTO-Tango screen,^23^ we did not include it here. Such discrepancies may be due to the assay differences. Notably, the PRESTO-Tango assay differs from radioligand binding assays in that it relies on beta-arrestin recruitment which is the final step of signal transduction and amplification. Therefore, higher receptor density or prolonged receptor signaling is needed to detect agonism,^43^ which may not have been achieved with all GPCR–chemical interactions. Indeed, EC_50_ values for BPA activation of DRD1 and 5HT2C in radioligand binding assays are double^41^ the highest concentration tested in the primary screen. This implies that BPA and DEP should not be eliminated as potential agonists of the receptors investigated here.

In summary, the primary screen indicates that chemicals present in a range of plastic FCA may activate certain GPCRs. To confirm the robustness and biological activity of these hits, we performed a secondary screen by applying a dose–response design.

### 3.3 ADORA1, MC3R, MTNR1A, and MTNR1B Are Confirmed Targets of Plastic Chemicals

**Figure 2:**
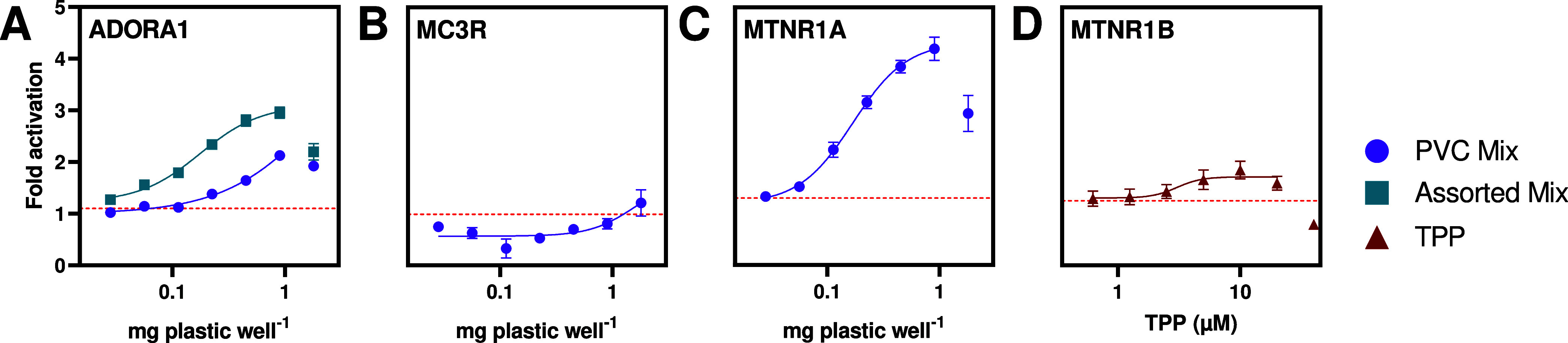
Secondary PRESTO-Tango screen confirms four (A, C, and D) and tentatively confirms one (B) out of 11 GPCR–chemical interactions. The highest concentration was, in some cases, excluded from the dose–response curves due to its sharp decrease in activity. The red horizontal line represents the LOD. Data are shown in mean fold activation ± SEM of three biological replicates with four technical replicates each.

Of the 11 hits, we confirmed four receptor–chemical interactions in the secondary screen: the PVC mix activated ADORA1 and MTNR1A in a dose-dependent manner, the assorted mix did the same at ADORA1, and TPP is an agonist of MTNR1B (Figure 2). This is, to the best of our knowledge, the first work demonstrating that real-world plastic products contain GPCR agonists that activate these receptors. The activity of TPP on the MTNR1B receptor has also not been previously reported.

Chemicals present in the assorted mix activated ADORA1 more effectively (3-fold change, EC_20_ = 0.07 mg plastic well^–1^) as compared to the PVC mix (2.3-fold change, EC_20_ = 0.56 g mgplastic well^–1^, Figure 2A). In contrast to many of the GPCRs investigated here, ADORA1 has been previously screened as a target of exogenous chemicals.^41^ Using three radioligand binding assays, ToxCast identified 115 chemicals as active ADORA1 ligands, including certain phenols and phthalates found in plastics.^41^ In fact, 45 of these are plastic-related substances, some of which have known endocrine activity (Table S5).^19^ However, we did not detect these plastic-related chemicals in our samples,^28^ suggesting that the observed ADORA1 agonist(s) are previously unidentified.

The compounds in the PVC mix also induced MC3R activity >LOD at 1.8 mg plastic well^–1^ (Figure 2B). While this indicates that MCR3 agonists are present, we were unable to test higher concentrations due to cytotoxicity. Such limitations are common when working with complex mixtures as they can incur nonspecific cytotoxicity before any specific effect can be observed.^37^ Due to the weak response, we were only able to tentatively confirm the hit and did not conduct further experiments. Although we could not confirm the dose dependence of the effect of the chemicals present in PVC, they activated MC3R at high sample concentrations.

The chemicals in the PVC mix induced a 4.2-fold activation of MTNR1A with an EC_20_ of 0.08 mg plastic well^–1^ (Figure 2C). Within the same GPCR family, TPP caused a 2-fold activation at MTNR1B (EC_20_ = 2.19 μM), though the activity decreased at higher concentrations either due to the promiscuous inhibitory effect of TPP observed in the primary screen (Figure 2D) or onsetting cytotoxicity (Figures S4 and S8). Curiously, PVC mix and TPP did not act similarly on the two melatonin receptors, despite their shared endogenous ligands and conserved orthosteric binding sites.^44^ Each receptor selectively binds unique ligands^45^ and differentially recruits beta-arrestin for the same compound,^46^ suggesting that the chemicals in this study may act as receptor-selective agonists. While plastic chemicals have not previously been described to target MTNR1A or MTNR1B, several carbamate insecticides with high structural similarities to melatonin and can agonize or antagonize both receptors.^10,11^

We did not confirm the following hits, as they did not produce a dose–response relationship in the secondary screen: PVC mix at prostaglandin E receptor 1 (PTGER1), assorted mix at prostaglandin E receptor 3 (PTGER3), PET mix at AVPR2, PET mix at lysophosphatidic acid receptor 5 (LPAR5), and TPP at apelin receptor (APJ, Figure S9A–F). The decreasing relationship for certain interactions (assorted mix at PTGER3) suggests that nonspecific effects are partially or fully masking activation of the receptor, as discussed above in the case of MC3R. In other cases, activation may be caused by a weaker secondary response. For example, the activity of the assorted mix on PTGER1 (Figure S9C) may be an indication of a general cellular stress response producing endogenous prostaglandins, which then act on PTGER1 in a para- or autocrine manner.^47^

Although only four of the hits from the primary screen could be validated, a subset, particularly ADORA1 and MTNR1A, exhibited robust confirmation. While high throughput assays are susceptible to both false positives and negatives,^48^ parallel screening facilitates identification of frequent hitters, leaky luciferase expression, and independent activation of the reporter.^23,30^ Therefore, the unconfirmed hits in the present study warrant further investigation to ascertain whether they are true false positives or a sign of more complex receptor–chemical interactions.

### 3.4 PVC and PUR Products Contain Potent and Effective GPCR Agonists

Given that the assorted and PVC mixes produced the most robust hits in the secondary screen, we investigated the activity of the individual extracts constituting those mixes. Accordingly, we tested the three extracts in the PVC mix on ADORA1 and MTNR1A and the eight extracts in the assorted mix on ADORA1. In this manner, we identified specific products and polymer types that contained the respective GPCR agonists.

**Figure 3:**
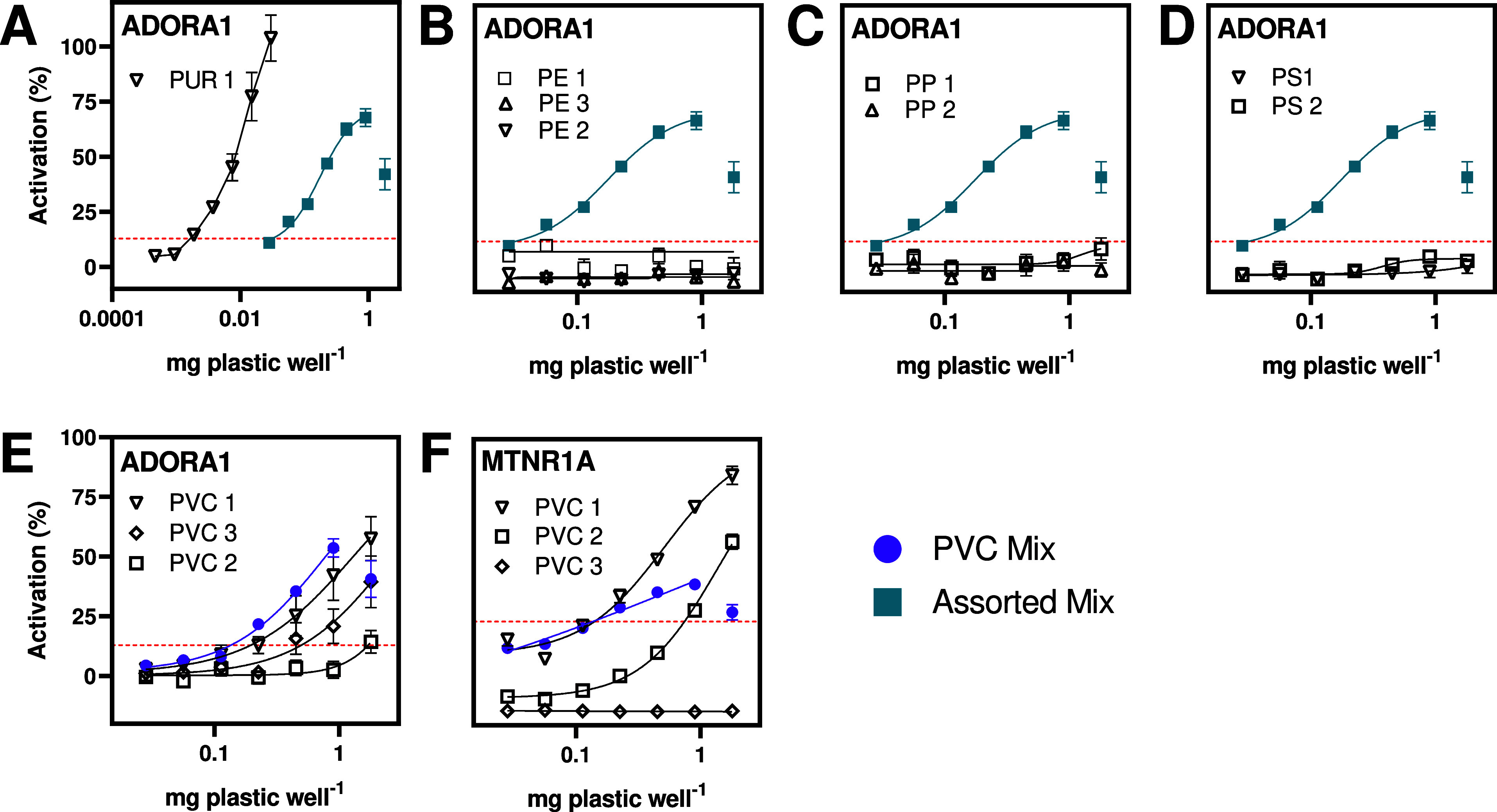
Activity of individual plastic extracts used in the assorted mix (blue) on ADORA1 (A–D) and in the PVC mix (purple) on MTNR1A (E, F). The concentrations of assorted and PVC mixes correspond to 1/8 and 1/3 of the concentrations of individual extracts, respectively. Data are shown as mean ± SEM of three biological replicates with four technical replicates each and were normalized to the maximal activity of the reference compound and the CA of the receptor. The red dotted line denotes the limit of detection.

Of the six polymer types included in our study, only products made of PVC and PUR contained GPCR agonists that induced dose-dependent activity (Figure 3). For ADORA1, PUR 1 (a drinking tube) was, by far, the most potent and effective extract inducing a 104% activation at the highest noncytotoxic concentration (EC_20_ = 0.005 mg plastic well^–1^, Figure 3A–D). PUR 1 was the only active extract in the assorted mix and contained 1785 unique features that were not present in any other sample in that mix (Figures S10 and S11). Identification of potential active chemicals is challenging as only 14% of features could be tentatively identified. Among them, that are most abundant and likely to be used in plastics are octrizole (CAS 3147-75-9) and TPP;^28^ however, the latter can be ruled out based on the results of the primary screen.

While we conclude that PUR contains chemicals that act as ADORA1 agonists, the lower maximum activity of the assorted mix compared to PUR 1 (Figure 3A) also points toward the presence of chemicals in the other polymer types that suppress this agonistic effect. Accordingly, receptor activity can be masked by GPCR antagonists or other inhibitors present in plastics. This demonstrates that mixture toxicity plays a critical role when testing complex mixtures, an aspect which becomes even more complex considering that GPCRs mediate signals both orthosterically and allosterically, often in tandem.^49^

Chemicals present in all three PVC products activated ADORA1, albeit with differing efficacies and potencies (Figure 3E). PVC 1 was the most active (58%, EC_20_ = 0.35 mg plastic well^–1^), followed by PVC 3 (40%, EC_20_ = 0.79 mg plastic well^–1^) and PVC 2 (14%, EC_20_ = 2.18 mg plastic well^–1^). This shows that ADORA1 agonists are abundant in PVC products. As only 4% of chemical features were shared by all PVC samples, it may be different chemicals in each sample causing the observed activity (Figures S10 and S11). Indeed, nontarget chemical analysis demonstrated large variation in the presence and abundance of features between samples of the same polymer type and product.^28^

For MTNR1A, PVC 1 was also the most active (85%, EC_20_ = 0.18 mg plastic well^–1^) followed by PVC 2 (57%, EC_20_ = 0.52 mg plastic well^–1^). These samples shared 297 features (Figures S10 and S11), of which 38 were tentatively identified.^28^ However, as none of these have been previously identified as MTNR1A agonists, elucidating the active chemicals will require more in-depth studies. Interestingly, PVC 3 reduced the MTNR1A activity to approximately 20% below CA at all concentrations (Figure 3F), suggesting the presence of inverse agonists, antagonists, or other inhibitors in the extract. This is further supported by the comparably lower activation caused by the PVC mix than that by its individual components.

The screening of individual plastic FCAs enables the identification of polymer types containing GPCR agonists and may provide a solution to minimize human exposure to such chemicals, regardless of their identity. For example, it is well established that PUR and PVC are the most problematic polymers due to the use of hazardous chemicals^50^ that induce a wide array of toxic effects including endocrine disruption.^27,39,51^ In addition, they may be more prone to leaching chemicals due to their amorphous polymer structure. Nonetheless, both materials are still used in some FCA, such as water pipes and, more commonly, in consumer products, among them children’s toys.^27^ This study reinforces existing evidence that PUR and PVC plastics are chemically problematic and should be substituted with safer alternatives.

### 3.5 Confirmation of Receptor Specificity

**Figure 4:**
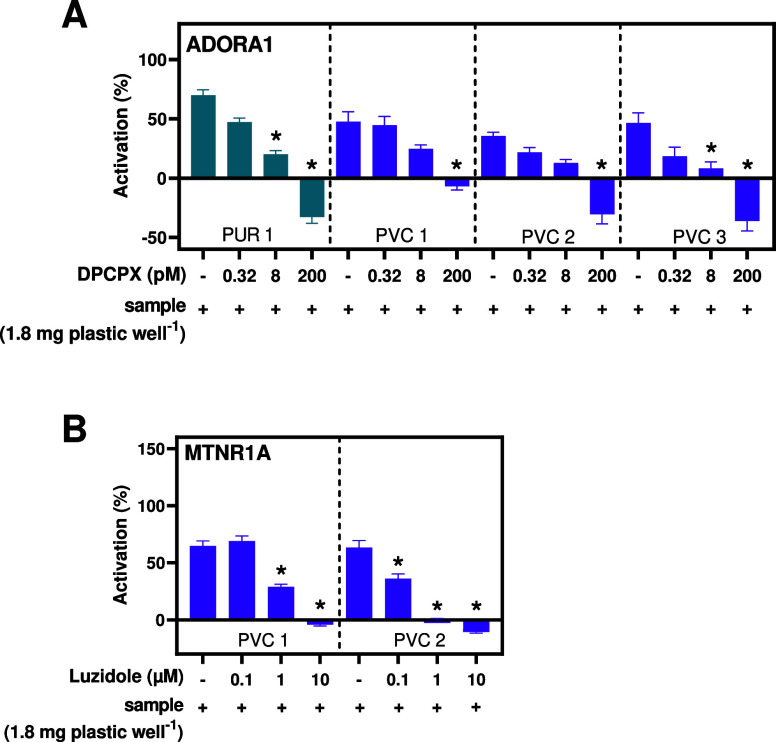
Chemicals in PVC and PUR are competing for binding sites at (A) ADORA1 and (B) MTNR1A with known receptor antagonists. Coexposure with DCPCX and luzindole significantly reduces the activation of ADORA1 and MTNR1A, respectively. Data are shown as mean ± SEM of three biological replicates with four technical replicates, each, and normalized to the maximal activity of the reference compound (100%) and the CA (0%) of the receptor. Asterisks indicate significantly different activity from the control (*p* < 0.05, one-way ANOVA between the control (−)).

To further confirm receptor specificity of the activity induced by plastic chemicals, we performed a pharmacological knock-down with known antagonists of ADORA1 and MTNR1A (dose–response relationships in Figure S12). Coexposure with the active PVC and PUR extracts and the ADORA1 antagonist DCPCX significantly reduced the activity induced by the reference compound (NECA), PUR 1, and all PVC extracts in a dose-dependent manner, in line with their potency (Figure 4A). Similarly, coexposure with the MTNR1A antagonist luzindole suppressed the activity of PVC 1 and 2 in a dose-dependent manner (Figure 4B). The highest concentration completely knocked down the effect of both extracts, and again, the more potent PVC 1 extract required higher concentrations of luzindole to reduce its activity. These experiments demonstrate that the well-known ADORA1 and MTNR1A antagonists compete for the same receptor binding sites as the compounds present in PVC and PUR products. We, thus, further confirm that these products contain melatonin or adenosine receptor agonists.

### 3.6 Biological Implications

Given that plastic chemicals are binding and activating GPCRs, we strove to understand the implications for GPCR signaling and, further down the line, the potential impacts on human health. To do so, we used biological process GO terms annotated to ADORA1 and MTNR1A. The relevance of each annotation was determined by its biological specificity, as quantified by their hierarchical level in relation to the maximal attainable level within the GO–DAG. Given that higher level GO-terms relate more specific biological information,^35,52^ this allows for a more precise characterization of a gene’s function within that process, and therefore, more accurate interpretations of potential biological consequences of GPRC activation.

ADORA1 had 45 annotations after elimination of GO-terms that shared GO–DAGs (Figure S13 and Table S6). These terms describe a broad spectrum of biological processes, underscored by the receptors extensive distribution and distinct functionality within various tissues.^53^ Conversely, MTNR1A had five unique annotations. The specific GO term “negative regulation of circadian sleep/wake cycle, non-REM sleep” (*s*_t_, 0.53) annotated to ADORA1 converges into the same GO–DAG of “circadian rhythm” (*s*_t_, 0.02), annotated to MTNR1A. Indeed, both ADORA1^54^ and MTNR1A^55,56^ regulate and maintain circadian systems that align cellular, physiological, and behavior processes into a 24-h cycle. Disruption of ADORA1 signaling is linked to alterations in sleep,^57,58^ an effect most people have experienced through the consumption of caffeine, an ADORA1 antagonist.^59^ Likewise, melatonin, or its synthetic mimics, are commonly prescribed to promote sleep via MTNR1A.^55^ Interestingly, exogenous melatonin can also promote sleep via adenosine receptors by inducing endogenous adenosine production.^60^ This exemplifies the importance of considering mixture toxicity, as multiple chemicals, with distinct biological targets, may regulate the same process to amplify disruption within an organism. While our work concerns only molecular initiating events, alterations of circadian homeostasis are clearly linked to serious human health impacts, including cancer,^61^ infertility,^62,63^ and impairment of immune function.^64^

The most specific GO term annotated to MTNR1A was “regulation of insulin secretion” (*s*_t_, 0.53). In addition to circadian regulation, MTNR1A receptor signaling has been associated with insulin sensitivity and the accumulation of fat, contributing to obesity and diabetes.^65,66^ Along the same lines, ADORA1 is largely expressed in white adipose tissue and regulates triglyceride homeostasis, fatty acid homeostasis, and lipid catabolic processes (Figure S13 and Table S6). We have previously shown that chemicals in PVC and PUR products increase adipocyte size and triglyceride content via an unknown PPARγ-independent mechanism.^38^ Given that the stimulation of ADORA1 in white adipose tissue increases adipogenesis,^67^ this could potentially represent a new mechanism that mediates the metabolism-disrupting effects of chemicals in plastics.

These results can be used to generate hypotheses on the downstream effects of plastic chemical disruption of ADORA1 or MTNR1A. However, such inferences are substantially complicated by the complexity of GPCR signaling. For instance, stimulation of ADORA1 in the brain reduces body weight and lipolysis^67^ but has the opposite effect in adipocytes. Although acting on the same receptor, an organ-specific distribution of both the receptor and the chemical could elicit contrasting adverse outcomes. As another example, prolonged exposure to MTNR1A agonists can reduce receptor density resulting in antagonistic effects.^68^ In addition, bias signaling, homo- or heterodimerization, and allosteric binding^69^ must be given consideration when extrapolating from in vitro to in vivo systems. Nevertheless, the potential link between metabolic and circadian disruption mediated through ADORA1 and MTNR1A warrants follow-up research.

### 3.7 Limitations and Future Directions

In this work, we show that plastic FCAs contain potent GPCR agonists using multiple layers of evidence (replication, dose-dependency, and pharmacological knock-down). This is significant because, historically, research has largely focused on chemicals acting via nuclear receptors^70^ and overlooked GPCRs as targets. Our work addresses this blind spot and highlights the need to expand our focus to include a broader range of receptors.

While primarily designed for drug discovery, the PRESTO-Tango assay is a powerful tool for toxicological research. However, given that drug screens are designed to search for very potent agonists, it may lack the sensitivity to detect compounds with weak GPCR activity. While this work focused exclusively on agonist activity on GPCRs, we found evidence for the presence of antagonists within the plastic samples that may mask agonist activity, thereby increasing the rate of false negatives. Accordingly, future research should also include GPCR antagonism, for which the PRESTO-Tango assay is capable of screening, albeit at a lower throughput, as each receptor needs to be screened with its known agonist. Further, the assay can be optimized for specific receptors of interest, in both agonist and antagonist modes, however, our main aim was to identify the most robust GPCR-chemical interactions across many receptors in a high-throughput, simultaneous fashion.

In this manner, we were able to show that chemicals in plastic food packaging act as agonists of ADORA1 and MTNR1A. Three lines of follow-up research arise from this: first, we investigated all extractable chemicals from plastic FCAs using methanol. To investigate the potential of human exposure more closely, migration studies using more realistic food simulants, such as water and ethanol, can help to assess the leachability of GPCR agonists from plastics. Second, effect-directed analyses can be applied to identify the active ADORA1 and MTNR1A compounds. While this is not without challenges, such identification would facilitate replacing the active chemicals in PUR and PVC. Third, an in vivo assessment of the effects on circadian and metabolic processes caused by ADORA1 and MTNR1A activation is required to understand how our in vitro findings translate to more complex biological systems.

While in an early stage of discovery, our work adds GPCRs to the lists of molecular targets that can be disrupted by plastic chemicals. Given that melatonin is an integral part of the hormone system, MNTR1A disruption illustrates that EDCs can act via cell surface receptors as well. However, GPCRs not directly connected to the endocrine system should not receive less attention, as exemplified by the ADORA1 agonists present in plastic. Here, insights from pharmacological research can be used to prioritize GPCRs as important drug targets for further toxicological research.

In a broader context, this study contributes to the growing evidence that plastic products contain compounds or mixtures of compounds that elicit a diverse range of toxic effects. A fundamental reconsideration and redesign of the way we make and use plastics are imperative if plastics are to be considered safe. By adopting strategies that reduce the number and hazard of chemicals used in plastics, we can minimize exposure and reduce their contribution to the burden of disease.

## Supporting Information Available

Supporting methods and materials: transfection efficiency and immunostaining, cell profiler analysis, cell viability, PRESTO-Tango assay, activity of individual plastic extracts and data analysis; Figure S1, schematic of items and mixtures that constitute each mix included in the study; Figure S2, dose–response relationship for three procedural blanks at the (A) ADORA1 and (B) MTNR1A receptors; Figure S3, receptor expression for a selection of GPCRs; Figure S4, cell viability of nontransfected cells and transfected cells; Figure S5, cytotoxicity of plastic extracts, mixes, and bisphenol A (BPA), diethyl phthalate (DEP), and triphenol phosphate (TPP); Figure S6, dose–response relationship for (A) ADORA 1 exposed to reference compound NECA, (B) MTNR1A, and (C) MTNR1B exposed to reference compound melatonin; Figure S7, cytotoxicity of nontransfected HTLA cells; Figure S8, plate layout for the PRESTO-Tango primary screen; Figure S9, the secondary PRESTO-Tango screen hits that could not be confirmed in a dose dependent manner; Figure S10, clustered heatmap of chemical features included in the PVC mix, the PET mix, and the assorted mix; Figure S11, overlap of chemical features (%) between extracts that were combined to make the (A) assorted mix, (B) PVC mix, and (C) PET mix; Figure S12, dose–response relationship for (A) ADORA1 and (B) MTNR1A exposed to reference antagonists DCPCX and luzindole; Figure S13, biological processes regulated by ADORA1 and MTNR1A (PDF)
Supporting tables: Table S1, corresponding sample names to Stevens et al. 2023;^28^ Table S2, 126 GPCRs included in the primary screen; Table S3, positive control criteria of each step of the screen and subsequent experiments; Table S4, fold activation and *z*-scores for the 126 GPCRs in the primary screen (PRESTO-Tango assay); Table S5, active ADORA1 compounds (ToxCast) also classified as plastic-related substances; Table S6, GO terms with *s*_t_ scores for ADORA1 and MTNR1A (XLSX)

## Notes

The manuscript has been published as a preprint on bioRxiv (doi: 10.1101/2023.10.04.560665).
